# The expected and unexpected benefits of dispensing the exact number of pills

**DOI:** 10.1371/journal.pone.0184420

**Published:** 2017-09-19

**Authors:** Carole Treibich, Sabine Lescher, Luis Sagaon-Teyssier, Bruno Ventelou

**Affiliations:** 1 Département AMSE, Aix-Marseille Université, CNRS, EHESS and Centrale, Marseille, France; 2 SESSTIM, Sciences Economiques & Sociales de la Santé et Traitement de l'Information Médicale, Aix Marseille Université, INSERM, IRD, Marseille, France; 3 ORS PACA, Observatoire Régional de la Santé Provence Alpes Côte d'Azur, Marseille, France; Universita degli Studi di Ferrara, ITALY

## Abstract

**Background:**

From November 2014 to November 2015, an experiment in French community pharmacies replaced traditional pre-packed boxes by per-unit dispensing of pills in the exact numbers prescribed, for 14 antibiotics.

**Methods:**

A cluster randomised control trial was carried out in 100 pharmacies. 75 pharmacies counted out the medication by units (experimental group), the other 25 providing the treatment in the existing pharmaceutical company boxes (control group). Data on patients under the two arms were compared to assess the environmental, economic and health effects of this change in drug dispensing. In particular, adherence was measured indirectly by comparing the number of pills left at the end of the prescribed treatment.

**Results:**

Out of the 1185 patients included during 3 sessions of 4 consecutive weeks each, 907 patients experimented the personalized delivery and 278 were assigned to the control group, consistent with a 1/3 randomization-rate at the pharmacy level. 80% of eligible patients approved of the per-unit dispensing of their treatment. The initial packaging of the drugs did not match with the prescription in 60% of cases and per-unit dispensing reduced by 10% the number of pills supplied. 13.1% of patients declared that they threw away pills residuals instead of recycling—no differences between groups. Finally, per-unit dispensing appeared to improve adherence to antibiotic treatment (marginal effect 0.21, IC 95, 0.14–0.28).

**Conclusions:**

Supplying antibiotics per unit is not only beneficial in terms of a reduced number of pills to reimburse or for the environment (less pills wasted and non-recycled), but also has a positive and unexpected impact on adherence to treatment, and thus on both individual and public health.

## Background

European countries vary in their methods of supplying drugs, some dispensing packaged drugs (such as Belgium, Austria, Sweden, Italy or France), others dispensing the exact number of tablets required (the Netherlands, the UK or the Czech Republic). The healthcare system, and in particular the method of supplying antibiotics, has been shown to impact the risk of misuse of drugs, for example through self-medication [1]. Yet, to the best of our knowledge, a proper comparison between these two dispensing systems has not been undertaken.

The recent and sustained development of bacterial resistance [2] has prompted many European countries to combat this major public health problem. France has high indicators of antibiotic resistance in numerous domains (e.g.: staphylococcus pneumonia, with tested susceptibility to penicillin or macrolides) [3], which can be explained by the very high prescription rates for antibiotics and plausible overuse or misuse of these drugs [4]. After a decrease in the early 2000s, France recently experienced an increase in antibiotic consumption [5]. While the pharmaceutical industry usually argues that packaging ensures traceability and provides better patient information and safety, this dispensing mode has the disadvantage of not always matching the prescription. This can lead to losses for the national healthcare system, misuse of antibiotics—for example, self-medication or overlong treatment-, as well as surplus pills being released into the environment. On the level of society as a whole, dispensing the exact number of pills required by the medical prescription may be one way to avoid these potential effects. However, on the individual level, patients may resist changes in the mode of dispensing, may feel they are losing out without the usual packaging and may react negatively, for example in terms of adherence to treatment.

Through a controlled intervention among community pharmacies over one year, we assessed the feasibility and the real impact of a change in the method of dispensing antibiotics in French community pharmacies for 14 antibiotics (see list in the Appendix A). This article reports the first results of this experimental intervention study. Assessed outcomes were: number of pills saved, potential gains for the environment and adherence to treatment.

## Methods

### Study design

Throughout 2015, a clustered randomized controlled trial was implemented in 100 retail pharmacies selected from the 161 that volunteered to participate, as invited through a call for applications by the Regional Health Agencies (ARS) in each of the study’s four regions. Pharmacies were allocated, based on a random number, in a 1:3 control-to-case ratio design according to three strata: regions (using the French administrative borders: Ile-de-France, Limousin, Lorraine, Provence-A1pes-Côte d’Azur), population density (rural/ urban) and level of activity (above and below median revenues). Within each strata, we assigned a random number to each pharmacy using the RAND function in Excel. Pharmacies were ranked based on this number and the first quartile was allocated to the control group and the others to the intervention group. The 100 pharmacies were compensated financially for their participation. 25 pharmacies (control group) continued providing antibiotics in the usual standard packaging from pharmaceutical companies, while 75 pharmacies (intervention group) dispensed the exact number of pills to patients with a prescription for the eligible antibiotics.

### Sample size computation

At the time of the protocol deposit, there was still some uncertainty regarding the number of participating pharmacies, and thus on the number of clusters. For this reason, a sample size pre-calculation was not possible. Fernandes and al. [6] studied adherence behaviour in Portugal with roughly 350 outpatients recruited in community pharmacies. In order to be on the safe side, we estimated that 700 subjects were needed to estimate a significant difference in the main outcome variables that we intend to deal with. In the course of the experiment, we were able to increase the sample size up to 1185 respondents.

### Data collection

This design was coupled with an observational study of pharmacists, to assess the percentage of cases where the prepacked boxes were not matching the prescribed number of pills as well as, in each case, the precise number of pills supposedly wasted if pre-packed boxes were used. The pharmacists recruited in this experiment were given study-related training: understanding of the protocol, rules for data collection, and modalities of patient recruitment. During 3 sessions of 4 consecutive weeks each, the protocol required pharmacists to recruit patients in a systematic way. First, they asked all patients with an antibiotic prescription whether they agreed with unit dispensing. Then, whatever the response, they invited patients to participate in a telephone survey. Participants were unselected (no age limit, for instance), except people who came to buy a treatment for a third party, who were excluded. Out of the 1,731 recruited by pharmacy staff, 1,238 patients actually agreed to be interviewed by phone by independent interviewers two to three days (on average) after completion of their treatment (attrition rates are equivalent in the two arms -T-test of differences with p-value = 0.34). In addition to questions about acceptance of the dispensing mode, the research team collected data on habits of recycling drugs, intended self-medication, quality of the information provided by the pharmacy staff for the current antibiotic treatment and adherence to this treatment.

### Main outcome variables

Outcome variables relate to economic concerns: the number of pills saved in the per-unit delivery mode; but also to social and public health benefits: acceptance from patient; quality of information received at the pharmacy; adherence behaviour. To this end, respondents were questioned retrospectively about the whole course of their antibiotic treatment, from pharmacy dispensing, to end of treatment (generally at home). First, the number of pills they received, together with the medical prescription, was recorded by the pharmacist. Second, the number of pills left after completion of treatment was collected through the patient’s survey. Using information on the number of pills provided by the pharmacies, we are able to compute the number of pills left in the two arms of the study, for a comparative purpose. For patients in the control group, the information was corrected in order to exclude the useless extra pills received by those patients; e.g., for one patient receiving a box of 30 pills for a treatment of 3 pills per day for 8 days (a real need of 24), we systematically correct his declaration of pills-left after completion by 6 (30–24) which was an extra amount NOT relevant for the comparison. We then inferred the patient’s “adherence” by comparing the expected and the actual number of pills left after completion of treatment (a similar counting method was used, for example, by [7] to measure adherence to antibiotic treatments in outpatients’ daily life).

Finally, a standardized and validated scale [8] of adherence, named the Morisky scale, was introduced in the questionnaire. From all this, we defined three distinct indicators of patients’ adherence: no pills left (strict adherence criterion), less than four pills left (one-day tolerance criterion) and a mixed indicator using both pill counts and self-declared scale (mixed adherence criterion).

### Data analysis

The randomization of pharmacies ensured not only the comparability of patients’ individual characteristics in the two arms, but also unbiased assessment of the impact of the change in antibiotic dispensing mode. We checked the quality of the randomization by T-test.

The outcomes described above (number of pills supplied, quality of information received at the pharmacy) are compared, for the two arms of the intervention by descriptive statistics and T-test. The adherence outcome is compared through a probit regression with robust standard errors clustered at the pharmacy level and sample selection equation (Heckman). This identification strategy allows ensuring that there is no sample-effect.

### Ethics and trial registration

The study is not a classical clinical trial, i.e. competition between different types of drugs—with possible severe impact on the health of the patient, but a simple modification in the delivery mode of the same drug. In that case, the French law does not constrain to register the study as a clinical trial. For this reason, the stakeholders of the study (the French Ministry of Health, unions of pharmacists and the scientific research team) asked for an approval of the ethic committee rather that a trial registration. Ethics Evaluation Committee of Inserm (the French National Institute of Health and Medical Research, CEEI) has delivered a favourable opinion on November 13^th^, 2014 (opinion 14–185). Furthermore the agreement of the CNIL (the French National Commission for Data Protection and Liberties) regarding the research protocol has been obtained on October 3^rd^, 2014. Finally, The Advisory Committee on Information processing regarding Medical Research (CCTIRS) has delivered a favourable opinion on January 15^th^, 2015. All patients receiving the exact number of pills required signed an informed consent certifying they agreed on the dispensing mode of their treatment. All patients interviewed signed an additional consent to indicate that they agreed on the telephone survey participation.

## Results

### Per-unit dispensing acceptance

The patient acceptance rate for per-unit dispensing was 80.6% (computed over the whole sample of eligible patients, thus eliminating any possibility of bias from agreeing to the phone survey). Of eligible patients, 39.3% agreed to take part in the phone survey. Individuals who refused the per-unit dispensing were excluded from the analysis living us 1,185 out of the 1,238 interviewed participants (see flow chart in appendix B).

### Randomization check

A first validity test of the randomization is to verify the absence of differences in observable characteristics between the participants in the two groups. Results are reported in Table 1.

**Table 1:** Patient and community pharmacy characteristics.

	Control group	Treated group	
Variables	% (X/N) [SE]	%, (X/N) [SE]	p-value
*Randomization at patient level—excluding individuals who refused per-unit dispensing*			
Female gender	60.8 (169/278)	62.7 (569/907)	0.56
Age of adult patients (in years) ¤	52.8 (246) [17.0]	54.3 (862) [17.0]	0.22
Low socio-economic status	52.9 (129/244)	50.2 (392/781)	0.47
Has children	81.8 (224/274)	81.4 (728/894)	0.91
Married	59.3 (162/273)	54.3 (483/890)	0.14
Employed	53.1 (145/273)	49.1 (434/884)	0.25
Chronic disease	48.9 (135/276)	46.0 (410/892)	0.39
*Randomization at community pharmacy level*			
Ile de France	20.0 (5/25)	19.2 (14/73)	0.93
Limousin	32.0 (8/25)	32.9 (24/73)	0.94
Provence-Alpes-Côte d’Azur	20.0 (5/25)	19.2 (14/73)	0.93
Lorraine	28.0 (7/25)	28.8 (21/73)	0.94
Length of time under current ownership (in years) ¤	14.8 (24)	14.8 (68)	1.00
Carries veterinary supplies	29.2 (7/24)	33.8 (23/68)	0.68
Provides drugs to EHPAD (nursing homes)	41.7 (10/24)	30.4 (21/69)	0.32
Has a confidential space	79.2 (19/24)	84.1 (58/69)	0.59
Agrees to disclose pharmacy turnover	76.00 (19/25)	78.1 (57/73)	0.83
Annual turnover (including tax) ¤	1 573 586 (19)	1 670 005 (57)	0.63
Proportion of turnover in reimbursed drugs ¤	79.0 (20)	79.9 (58)	0.45
Number of full-time pharmacists ¤	4.8 (24)	5.0 (68)	0.89
Number of full-time pharmacy technicians ¤	1.6 (24)	1.9 (68)	0.32

Notes: Variables marked with ¤ are continuous. Figures in brackets are the raw number of cases (X) divided by the number of observations (N). For example, line 1: X = 169 females, among N = 268 patients, in the control group; X = 569 females, among N = 907 patients, in the treated group. Variations in the number of observations are due to missing data. When the patient was a child (77 cases), characteristics refer to parental data.

### Economic consequences

The initial drug packaging had to be modified in 60.0% of cases of per-unit dispensing, meaning that the packaging available in the community pharmacy did not match the prescription (or the reverse: the prescription did not match the pre-packed box). Dispensing the exact number of drugs reduced by 9.9% the number of pills supplied—in France, to be charged to the Social Security fund–, compared to the traditional delivery mode in pre-packed boxes. The average number of pills supplied was 23 pills when pills were delivered traditionally against 20 when delivered per unit (T-test of differences with p-value = 0.02).

### Information provided by the pharmacist

Respondents were asked whether the pharmacist who attended them provided a series of information regarding their treatment (treatment length, number of doses per day, recycling mode, risk of non-adherence, among others). No difference in information given to patients regarding appropriate use of their medication was found between the two groups—see Appendix C.

### Self-medication and recycling issue

In the phone survey, 17.6% of respondents declared their intention to keep the antibiotics left after completion of treatment. 10.7% of respondents also admitted that they could use antibiotics without the consent of a physician. The reduction in residuals induced by the per-unit delivery could lead to a decrease in self-medication of 1.9% (10.7% x 17.6%). Last, 13.1% reported that they generally throw away their pills instead of recycling (no difference between groups).

### Adherence

Per-unit dispensing also appeared to improve adherence to the antibiotic treatment. A patient was considered as “adherent” if he had no pills left at all (intervention group), and after excluding extra pills received due to a prescription / packaging mismatch (control group). The pill count information is available for 984 respondents. 65.6% and 91.4% of patients were adherent (strict adherence criterion) in the control and the intervention groups respectively (see Table 2).

**Table 2:** Descriptive statistics of adherence to treatment.

	Control group	Treated group	
Variables	% (X/N)	%, (X/N)	p-value
Strict adherence criterion	65.6 (84/128)	91.4 (782/856)	0.00
One day tolerance criterion	71.1 (91/128)	92.3 (790/856)	0.00
Mixed adherence criterion	57.5 (73/127)	77.8 (660/848)	0.00

Notes: Figures in brackets are the raw number of cases (X) divided by the number of observations (N). For example, line 1: X = 84 are adherent according to the strict adherence criterion, among N = 128 patients, in the control group.

From Table 3, presenting multivariate regressions, we see that the probability of being adherent is always greater in the intervention group. Marginal effects ranged from 0.23 [0.15; 0.31] to 0.21 [0.14; 0.28] depending on the measures (with 95% interval confidence in bracket). This first value of 0.23 means that patients under the per-unit delivery system are estimated 23 percentage-points more likely to be adherent to their antibiotic treatment. Robustness tests involved (i) taking a looser indicator of patient adherence: having less than a day’s medication left and (ii) combining the count with a standardized scale of adherence [8].

**Table 3:** Impact on adherence of dispensing exact quantities required.

	Strict adherence criterion (no pills left)	One day tolerance criterion (less than four pills left)	Mixed adherence criterion (counting method combined with the Morisky scale)
Treated	0.23*	0.20*	0.21*
Pharmacies	[0.15; 0.31]	[0.12; 0.29]	[0.14; 0.28]
Excluding individuals who refused per-unit dispensing	Yes	Yes	Yes
*Observations*	*1185*	*1185*	*1185*
*Censored observations*	*984*	*984*	*975*

*Notes*: Probit estimations, with sample selection equation (at first step, using Heckman command). Marginal effects reported. Robust standard errors clustered at pharmacy level. IC at 95% reported in brackets.

* p< 0.01.

Control variables are age and type of treatments; they were introduced after a stepwise procedure which determines their significance at the critical p-value of 0.10.

## Discussion

### Major findings

This study investigates the impact of per-unit dispensing of 14 antibiotics on three outcomes likely to generate antibiotic resistance: self-medication, surplus pills being released into the environment and compliance with treatment [3–4, 9]. We find all-round beneficial effects. First, lower quantities of antibiotics are supplied under the per-unit dispensing system. This confirms that, despite the claims of the pharmaceutical industry or of retail pharmacists, there is a degree of mismatch between prescriptions and packaging (this may be either due to the drug dispensing system or the physician, who may prescribe the wrong quantity or wrong treatment period). This mismatch suggests a potential source of savings for the French national healthcare system that reimburses prescribed medicine (as, for each pill delivered in excess in the traditional delivery mode, there is waste of financial resources—however see the dedicated paragraph below for possible counterbalancing limitations and costs). Second, the lower likelihood of having pills left at the end of treatment (often not recycled) may also reduce self-medication and bacterial resistance, consistent with findings by Grigoryan and co-authors [1]. Supplying antibiotics per unit might thus have long-term positive effects on the environment, although, because restricted to the (only) indirect evidence of probable release of pills in the nature, the design of the study does not permit us to assess these delayed effects in a precise quantitative manner (ecological indicators of bacterial resistance). Third, dispensing the exact number of pills needed appears to increase adherence to the treatment. Although the beneficial economic and environmental effects of this type of drug supply were anticipated, the improvement of adherence was not.

While pharmaceutical companies defend their pre-packed drug packaging, this does not always seem to be an efficient way of dispensing antibiotics, in economic, environmental and patient’s health terms. Note that the benefits from adherence can also be linked with parallel outcomes related to the population’s health: non-adherence to antibiotic treatment leads to therapeutic failure, re-infection and bacterial resistance [3, 4, 9].

### Underlying mechanisms

This study is one of the first to measure outpatient adherence to antibiotic treatment. While some researchers showed that factors related to the antibiotic itself, the patient and the patient-physician relationship influence adherence [6, 9], we demonstrate that the drug dispensing method also has an effect, likely “causal” through the design of the study. Plausible explanations include *cognitive simplification*: individuals who know that they have to finish their stock of pills have at their disposal a visible day-to-day treatment schedule (the end of the treatment is clearly indicated by the termination of the stock); *psychological support*: the fact that the pharmacist devotes scrupulous attention to repackaging and counting out the exact number of pills may incite the patient to complete the treatment more thoroughly.

### Adherence measurement limitations

We acknowledge that our measurement of adherence relies on patients’ declarations, which may be a limitation. While memory bias is limited due to the short lapse of time between completion of treatment and the interview, a social desirability bias may arise when patients declare how many pills they have left (more likely in the intervention group). However, only indirect measures are possible with outpatients and with such acute diseases. It might be possible, by observing data on health expenditure reimbursements, to identify the dispensing of an equivalent drug a few weeks after completion of the initial treatment (a second similar prescription could indicate poor compliance). Such data have not been obtained yet. Nevertheless, robustness tests combining two validated tools encourage some confidence in our results. Finally, the random design of the study should ensure that any measurement errors are equally distributed in the two experimental arms (for adherence behaviours and the other outcomes considered).

### Other limitations

Other weaknesses of the study were the following: participants were not asked about the diagnosis associated with the antibiotic prescription (this would imply a cross-validation by the physician which was difficult to set in place); the participation to the phone survey was disappointing (but equal in the two arms); delayed effects on the environment (bacterial resistance) are not assessable in this small scale study; and last, we had to renounce to make a standard cost-benefit evaluation study, because equilibrium prices, fees and costs appeared too complex to estimate in this preliminary phase of the policy.

### Beyond the study itself

The cost of a change in dispensing, from the current pre-packing in boxes to a per-unit system, lies in an increased workload for pharmacists, not evaluated in this study (since no adjustment for per-unit dispensing had been made to the drug provision chain, due to the small scale of the intervention). We recognize that the absence of a precise accountability of these labour costs is an obstacle for the assessment of the complete “financial” consequences of the policy. If this dispensing mode were scaled up throughout the French territory, drug prices -for the industry- and possible dispensing fees -for the pharmacist- would have to be (re)negotiated with the Public Authority (the compensation granted to pharmacies in this experiment was including the participation to an experimental design and cannot be used as a basis for a cost study). Such considerations are beyond the scope of this paper, but our findings suggest that the various benefits from the policy, in terms of public health and Social Security reimbursement, might create a “margin” to be redistributed to stakeholders.

## Supporting information

S1 Table Distribution of antibiotics delivered. (PDF)

S2 Table Information given to patient. (PDF)

S1 Fig Flow chart. (PDF)
