# Supplementary material for: The expected and unexpected benefits of dispensing the exact number of pills
Source: PLoS One. 2017 Sep 19;12(9):e0184420. doi: 10.1371/journal.pone.0184420 (PMC5604959; doi:10.1371/journal.pone.0184420)
Supplement: S1 Table — (PDF) [file pone.0184420.s001.pdf]

Table S1: Distribution of antibiotics delivered

Name of the drug	Raw number	Share
**Combination of amoxicillin and clavulanic acid**		
Amoxicillin-clavulanic acid	520	43.88
**Third and fourth generation of cephalosporines**		
Cefixime	102	8.61
Cefpodoxime	268	22.62
Cefotiam	7	0.59
**Fluoroquinolon**		
Ciprofloxacin	94	7.93
Enoxacin	1	0.08
Flumequin	0	0.00
Ofloxacin	63	5.32
Levofloxacin	53	4.47
Lomefloxacin	16	1.35
Moxifloxacin	13	1.10
Norfloxacin	40	3.38
Pefloxacin		
**Amphenicols**		
Thiamphenicol	0	0.00
**Not in the list ¤**	8	0.68
TOTAL	1 185	

Notes: This table displays the 14 antibiotics considered in the study; they were listed by experts from the French Ministry of Health as relevant to fighting antimicrobial resistance. When we introduced control for the "type of treatment", we created a multimodal variable, a, b, c: with a) Amoxicillin-clavulanic acid; b) Cefpodoxime; c) all others.¤ The antibiotics not in the list are the following: cefuroxime (2), clarithromycine (2), spiramycine (1) and roxithromycine (3). Pharmacists included them in the study by mistake.
