# Supplementary material for: The expected and unexpected benefits of dispensing the exact number of pills
Source: PLoS One. 2017 Sep 19;12(9):e0184420. doi: 10.1371/journal.pone.0184420 (PMC5604959; doi:10.1371/journal.pone.0184420)
Supplement: S2 Table — (PDF) [file pone.0184420.s002.pdf]

Table S2: Information given to patient

Knowledge of:	Pharmacies Total	Treated	Controls	p-value
Treatment length	92.8 (1049/1131)	93.2 (803/862)	91.5 (246/269)	0.35
Number of doses per day	92.1 (1042/1132)	92.8 (802/864)	89.6 (240/268)	0.08
Dose spacing	44.0 (499/1133)	43.7 (378/865)	45.2 (121/268)	0.68
Storage method	10.0 (114/1135)	10.4 (90/868)	9.00 (24/267)	0.51
Risks of non-adherence	14.7 (167/1133)	15.7 (136/868)	11.7 (31/265)	0.11
Recycling mode	12.9 (146/1134)	12.7 (110/868)	13.5 (36/266)	0.71
Safety Precautions	47.3 (534/1130)	47.8 (413/865)	45.7 (121/265)	0.55
Side effects	19.5 (221/1135)	20.4 (177/868)	16.5 (44/267)	0.16

Notes: We excluded individuals who refused per-unit dispensing. Raw numbers are indicated in brackets.
