# Supplementary material for: The expected and unexpected benefits of dispensing the exact number of pills
Source: PLoS One. 2017 Sep 19;12(9):e0184420. doi: 10.1371/journal.pone.0184420 (PMC5604959; doi:10.1371/journal.pone.0184420)
Supplement: S1 Fig — (PDF) [file pone.0184420.s003.pdf]

Figure S1: Flow chart

Control		Intervention
**Eligible patients (patients who came to the pharmacy with an antibiotic treatment prescription)**		
965		3518
	Total =4483	
**Patients who declared to the pharmacists to agree to participate to the phone survey**		
391		1340
	Total =1731	
**Patients who were actually interviewed by phone**		
289		949
	Total =1238	
**Patients who accepted (either hypothetically or actually) the per-unit delivery of drug**		
278		907
	Total =1185	
**Patients who answered to all questions (in particular the number of pills left)**		
128		856
	Total=984	
